# An uranyl sorption study inside functionalised nanopores

**DOI:** 10.1038/s41598-020-62792-4

**Published:** 2020-04-01

**Authors:** U. Pinaeva, N. Ollier, O. Cavani, E. Balanzat, M. Al-Sheikhly, T. L. Wade, M.-C. Clochard

**Affiliations:** 1 Laboratoire des Solides Irradiés CEA-CNRS-Ecole Polytechnique UMR7642, Institut Polytechnique de Paris, 91128 Palaiseau Cedex, France; 2 Laboratory for Radiation and Polymer Science, Department of Materials Science and Engineering A. J. Clark School of Engineering, University of Maryland, College Park, MD 20742-2115 USA; 3 CIMAP, CEA-CNRS-ENSICAEN UMR6252, 14050 Caen Cedex, France

**Keywords:** Chemistry, Materials science, Nanoscience and technology

## Abstract

Sorption mechanism of uranyl by poly(bis[2-(methacryloyloxy)ethyl] phosphate) (PB2MP) functionalised polyvinylidene fluoride (PVDF) track-etched membranes, PB2MP-g-PVDF, was investigated. It was found that uranyl sorption obeyed Langmuir isotherm model giving a maximum U(VI) membrane uptake of 6.73 *μ*mol g^−1^ and an affinity constant of 9.85 ⋅ 10^6^ L mol^−1^. XPS and TRPL measurements were performed to identify sorbed uranyl oxidation state and its environment. Uranyl was found to be mainly in its hexavalent state, *i.e*. U(VI), showing that the trapping inside the PB2MP-g-PVDF nanoporous membranes did not change the ion speciation. Two sorbed uranyl life-times (*τ*_1_ = 8.8 *μ*s and *τ*_2_ = 102.8 *μ*s) were measured by TRPL which pointed out different complexations taking place inside the nanopores. Uranyl sorption by PB2MP-g-PVDF membranes was also found to be pH dependent demonstrating the highest performance at circumneutral pH. In addition, TRPL was demonstrated to be not only a remarkable technique for U(VI) characterization, but also an alternative to voltammetry detection for trace on-site uranyl monitoring using PB2MP-g-PVDF nanoporous membranes.

## Introduction

The detection of uranium trace concentrations is of great importance due to its extensive use in nuclear power plants. According to estimations by the International Atomic Energy Agency (IAEA), the total generated nuclear power will rise in coming decades^1^,pp. 21. Simultaneously, nuclear power plant dismantling will require fast on-site monitoring of uranium in nearby water and soils. A variety of spectroscopic techniques such as atomic absorption spectroscopy (AAS), inductively coupled plasma optical emission spectroscopy (ICP-OES), mass spectroscopy (ICP-MS)^2^, and X-ray fluorescence spectroscopy (XFR)^3^ can be used to detect uranium content in water samples at lab-scale. However, all these classic techniques are not portable and thus, involve large sample campaigns, making fast on-site monitoring difficult. In addition they are complicated and of high cost.

To address fast and simple on-site analysis, we have recently proposed a portable electrochemical sensor for trace uranium detection in water^4^. These sensors are made of track-etched poly(vinylidene fluoride) (PVDF) membranes functionalised with poly(bis[2-(methacryloyloxy)ethyl] phosphate) (PB2MP) enabling efficient uranyl preconcentration thanks to their nanoporous structure. The limit of detection (LOD) was estimated to be 17 ppb (3*σ*/slope) with a quantification limit (LOQ) of 51 ppb. In aqueous solutions, the most thermodynamically stable form of uranium is a hexavalent state as uranyl cation, UO${}_{2}^{2+}$. Similar to other actinides, it has a tendency to form strong complexes with oxygen-containing ligands, among which hydroxides, carbonates and phosphates prevail in natural water. A. Sanding and J. Bruno have found that uranyl phosphate interaction dominates especially at neutral pH, even if existence of the very strong uranyl carbonate complexes is considered^5^. Indeed, Das *et al*.^6^ reported the efficient uranium preconcentration from seawater by poly(etylene glycol methacrylate phosphate) (PEGMP) macroporous membranes and suggested that UO${}_{2}^{2+}$ forms a complex with PEGMP units in 1:2 proportion. The property of UO${}_{2}^{2+}$ to bind with phosphate groups explain efficient uranyl preconcentration by PB2MP^7^. However, as the objective is to integrate these sensors in a portable device, a deeper understanding of the mechanism of metal ion sorption at solid-liquid interface inside the nanopores is required.

Time-resolved photoluminescence (TRPL) is a highly sensitive technique for the analysis of actinides such as uranium and lanthanides (Eu^3+^, Tb^3+^, *etc*.) in a wide range of concentrations^8^, which can meet the requirements of fast ultra-trace uranium detection. Besides its high sensitivity, TRPL gives both spectral (wavelength shifts, peak shapes) and fluorescence life-time, characterizing uranium species environment^9^ and allowing investigation of uranium speciation and complexation mechanisms. A few reports concerning TRPL measurements of uranyl-phosphate species were published by Scapolan *et al*.^10^ in blood serum and by Meca *et al*.^11^ in river sediments contaminated by phosphate ore. The state-of-the-art in TRPL for uranium speciation analysis at ultra-trace concentrations in solution is by remote determination *via* pulsed nitrogen laser, fiber optics and optrode^8,12^. The LOD of uranium in 1 M phosphoric acid was found to be 24 *μ*g/L. Using fiber optics and a flow optrode, Wangen and co-workers^13^ were able to reduce even further the LOD over a wide range of uranium concentrations from 10^−4^ to 10^−9^ M.

In this paper, we investigated uranyl sorption behaviour of recently developed PB2MP-g-PVDF functionalised nanoporous membranes using Langmuir, Freundlich, and Temkin sorption isotherm models. To find out the mechanism of U(VI)-PB2MP underlying the sorption, batch sorption experiments were performed on PB2MP-g-PVDF membranes and compared with others metal ions/functional polymer complexes. Polyacrylic acid (PAA) and poly(4-vinyl pyridine) (P4VP) were grafted into similar track-etched PVDF membranes to efficiently trap Pb(II) and Hg(II) ions respectively. These two systems exhibit two very different well-known complexation states from a weaker affinity constant due to electrostatic interaction between Pb(II) ions and the weak acids of PAA up to a high affinity constant resulting in a strong metal/ligand interaction between Hg(II) ions and pyridine functional groups of P4VP. Finally, TRPL measurements were carried out and compared to XPS data to verify uranyl oxidation state and sorbed species environment.

## Experimental

### Materials

Semicrystalline *β*-poly(vinylidene fluoride) (PVDF) films of 9 *μ*m thickness were provided by PiezoTech SA. Potassium hydroxide, potassium permanganate, Mohr’s salt ((NH4)_2_Fe(SO_4_)2.6H_2_O), sodium metabisulphite (Na_2_S_2_O_5_), bis[2-(methacryloyloxy)ethyl] phosphate (B2MP), ethanol were purchased from Sigma-Aldrich. Uranyl acetate (UO_2_(CH_3_COO)_2_) was used for preparation of U(VI)-spiked aqueous solutions. Deionized water (resistivity 18 M*Ω*.cm) was used for preparation of all solutions.

### Functionalised track-etched PVDF membrane fabrication

#### Irradiation

Swift heavy ions (SHI) irradiations were performed at GANIL, France. PVDF films were irradiated at room temperature with _136_Xe^48+^ (7.46 MeV ⋅ amu^−1^) under He atmosphere. The films of 4 × 30 cm^2^ were anchored on movable sample holders and put inside the irradiation chamber. Ion fluence was adjusted taking into account number of passages and the ion beam flux. For all the samples, herein studied, the fluence was 10^10^ cm^−2^.

#### Track etching

SHI irradiated *β*-PVDF films were chemically etched using 10 M KOH and 0.25 M KMnO_4_ solutions at 65° C for 25 min^14^. The etched membranes were washed with aqueous solution of 7.5 wt.% Na_2_S_2_O_5_ to reduce the remaining *KMnO*_4_ followed by rigorous rinsing by deionized water. The diameter of obtained cylindrical nanopores depends on the etching duration. After 25 min of etching of 10^10^ cm^−2^ fluence films the resulting mean pore diameter was 60 ± 5 nm.

#### Radiation grafting

The track-etched PVDF membranes were placed in purgeable glass tubes containing 300 mM B2MP monomer solutions^4,15^. A mixture of ethanol and water in 1:1 ratio was used as a solvent. To avoid undesired homopolymerization reaction and limit grafting reaction to the nanopores, we followed pre-irradiation grafting method. However, Mohr’s salt (6.32 g/L) was added to every monomer solution prior grafting. The resulting track-etched PVDF membranes were immersed into monomer containing purgeable glass tubes and connected to a Schlenk line for oxygen elimination. After 15 min of N_2_ purging at room temperature, they were then sealed and put into a thermostat water bath at 65° C for 1 to 18 hours. The grafted membranes were washed with ethanol and dried at room temperature. Gravimetrical grafting yield (GY) was determined as the weight ratio of grafted PB2MP to initial PVDF membrane.The PB2MP-g-PVDF membranes with an optimal GY value of 4.5 wt.% were selected and used for all measurements in this work.

### Methods

#### TRPL measurements

TRPL spectra were recorded using a pulse Indi Nd: YAG laser (Spectra-Physics) of 1.5 mJ energy at excitation wavelength of 355 nm. Detection is performed using an intensified charge-coupled device (ICCD) camera coupled with an Oriel monochromator with a 150 l/mm grating associated to a Stanford delay generator. The gate width of 10 ms and gate delay of 400 ns were set.

For uranyl sorption, synthesized PB2MP-g-PVDF membranes were immersed into stirring U(VI)-spiked aqueous solutions of various concentrations for 22 hours. The sorbed membranes were then rinsed with deionized water to remove uranyl excess, blotted with a paper towel and kept in a plastic box. For TRPL measurements, each membrane was sealed in a quartz cuvette at a fixed position and put in the optical set-up.

#### Voltammetry

Voltammetry measurements were performed in a three-electrode electrochemical cell controlled by a PalmSens potentiostat (PalmSens, the Netherlands). A home-made inert clip with three inserted wires (two gold for membrane-electrode connections and one silver as a pseudo-reference) was used for membrane-electrode connection to a potentiostat. The PB2MP-g-PVDF membranes were converted into electrodes by depositing 35 nm of gold layers of 0.502 cm^2^ on each side of the membrane using a cathodic evaporator Quorum Q 150R S and a mask. A mixture of 50 mM acetate buffer and 0.1 M KNO_3_ (pH 3) was used as electrolyte.

#### Inductively coupled plasma mass spectrometry (ICP-MS)

An Agilent 7900 ICP-MS instrument (Agilent Technologies, Japan) equipped with a Scott quartz chamber and a quartz crossflow nebulizer for introduction of the analyzed solutions before and after uranyl sorption. The power of the plasma of 1550 W and nebulizer flow rate of approximately 1 L/min were used. Both U235 and U238 were analyzed and all measurements were performed in triplicate without collision/reaction gas.

For isotherm elaboration, previous kinetic experiments fixed the equilibration time at 17 h. Six various U(VI) concentrations of 10, 25, 50, 100, 500 and 1000 *μ*g/L were made from an uranyl acetate stock solution. The ICP-MS was calibrated prior injection series with different U(VI) solutions made from a stock standard solution (see SI-0). Initial U(VI) concentrations from uranyl acetate stock solution were then calibrated based on U238 number of counts. For each concentration, triplicate solid-liquid experiments were performed. Three different membranes were thus individually immersed in three vials containing U(VI) solutions of same initial concentration. Before and after membrane equilibrating, uranyl concentration of the solution was registered.

#### X-ray photoelectron spectroscopy (XPS)

XPS spectra were conducted on a PHI 5000 VersaProbe (Physical Electronics) instrument. An K-*α* (1486.6 eV) monochromatic X-ray source was used^4^. A 187.85 eV pass energy value was used for survey spectra and 23.5 eV for high resolution (HR) peaks. Charge compensation was accomplished with a combined electron and Ar neutralizer system. The binding energies were calibrated by fixing the C 1s binding peak to 284.5 eV.

## Results and discussion

### Functionalised membrane fabrication and characterization

**Figure 1:**
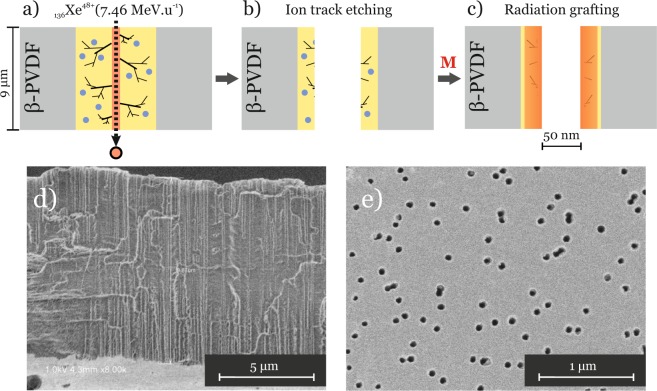
Overall schematic fabrication procedure of functionalised 9 *μ*m thick *β*-PVDF nanoporous membranes: (**a**) _136_*Xe*^48+^ irradiation at 7.46 MeV ⋅ amu^−1^ under He atmosphere. The black dashed arrow represents the pathway of an ion-projectile. It results in red (track core) and yellow (track halo) zones of radiation-induced defects; (**b**) nanopores formation by ion-track chemical etching; (**c**) radiation-grafting of B2MP monomer initiated by remaining intercrystalline trapped radicals from the nanopore walls; FESEM images of (**d**) cryofracture and (**e**) top view of a resulting functionalised membrane (fluence: 10^9^ cm^−2^).

The functionalised track-etched PB2MP-g-PVDF membranes were fabricated as described in^4^ and displayed in Fig. 1. PVDF films, -${[{CH}_{2} \mbox{-} {CF}_{2}]}_{n}$-, were first irradiated with _136_Xe^48+^ SHI of 7.46 MeV ⋅ amu^−1^ under He atmosphere. During irradiation, projectile-ions produce cylindrical damaged regions across the entire thickness of a PVDF film. These damages are radially distributed along the ion pathways forming so-called latent tracks. High energy radiation causes lateral bond and backbone chain scissions resulting in gas emission (H_2_, HF, F_2_), formation of alkyl radicals (–CF_2_–C.H_2_,–CH_2_–C.F_2_–CF_2_–C.H–CF_2_,–CH_2_–C.F–CH_2_), crosslinking, and unsaturated bonds (*e*. *g*. , − CF_2_ − , CH = CF_2_, − CH = CF − ). Despite the radiation process occurs under He atmosphere, some alkyl radicals located at the surface of the polymer film can be converted into peroxy radicals (ROO.) in contact with oxygen in further fabrication steps. To reveal the latent tracks into nanopores, the irradiated PVDF films were etched in 10 M KOH and 0.25 M KMnO_4_ solutions. As the relative radical fraction decreases as etching time increases, the etching reaction duration was limited to 30 min leading to nanopore size of around 30 nm of radius in order to preserve a significant density of radicals. Under these conditions, the remaining radicals were found sufficiently reactive to initiate a graft polymerization in presence of B2MP monomer. To efficiently graft B2MP monomer inside nanopores of the membrane, the grafting reaction temperature was kept at 65° C to activate trapped alkyl and peroxy radicals.

Equations (1–4) represent the radiation grafting mechanism accordingly to a free-radical polymerization. It consists of initiation, chain propagation and termination reactions.

1 $${\rm{R}}{\rm{H}}\mathop{\mathop{\to }\limits^{irradiation}}\limits_{\,He,\,roomT\,}{{\rm{R}}}^{\bullet }+{{\rm{H}}}^{\bullet }$$

2 $${{\rm{R}}}^{\bullet }+\,{\rm{M}}\,\to {{\rm{R}}{\rm{M}}}^{\bullet }$$

3 $${{\rm{R}}{\rm{M}}}^{\bullet }+\,n{\rm{M}}\,\to {{\rm{R}}{\rm{M}}}_{n+1}^{\bullet }$$

4 $${{\rm{R}}{\rm{M}}}_{n}^{\bullet }+{{\rm{R}}{\rm{M}}}_{m}^{\bullet }\to {{\rm{R}}{\rm{M}}}_{n+m}$$

**Figure 2:**
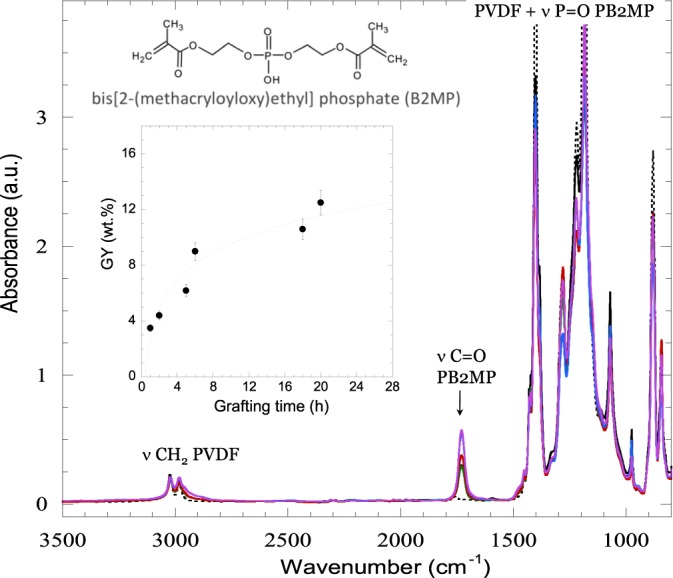
FTIR spectra of PVDF membranes before and after grafting with PB2MP at various radiation-grafting times: not grafted (black dot line), 1 h (black), 2 h (green), 5 h (red), 18 h (blue) and 20 h (purple). Inset: grafting time as a function of GY.

Functionalised PB2MP-g-PVDF membranes were characterized by FTIR (Fig. 2).

Together with absorption bands at 1211 and 1183 cm^−1^ corresponding to CF_2_, a doublet corresponding to the CH_2_ sym and asym vibrations at 2985 and 3025 cm^−1^ along with a multiplet with spectral absorption bands at 1453, 1424, 1403 and 1385 cm^−1^ which are usually attributed to a mixture of CH_2_ and C-C vibrations represent the footprints of PVDF. Below 900 cm^−1^, in the polymer skeleton vibration region, a well-defined peak at 840 cm^−1^ reveals the *β*-phase nature of PVDF crystallinity^16^. The absorption band at 1725 cm^−1^ corresponds to C=O stretching vibration of PB2MP. The stretching vibration of P=O that typically appears at 1350–1250 cm^−1^ is not observable due to overlapping with intense absorption bands of PVDF^17^. Increasing the grafting time resulted in higher grafting yields (Fig. 2, inset).

**Figure 3:**
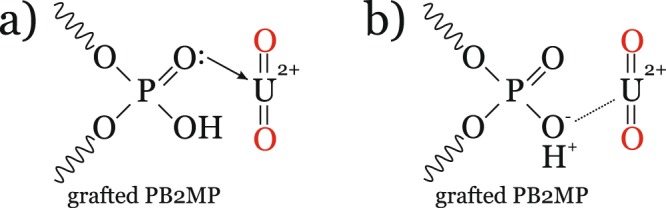
Schematic interactions involved in uranyl preconcentration by phosphate group of grafted PB2MP: (**a**) coordination and (**b**) cation-exchange or electrostatic interactions.

The PB2MP-g-PVDF membranes were then transformed into electrochemical sensors by a simple Au-sputtering on their surfaces and utilized for uranyl detection by means of cathodic stripping voltammetry (SW-CSV). After parameter optimization, a limit of detection (LOD) of these sensors was as low as 17 *μ*g L^−1^, or ppb^4^ showing the effective U(VI) trapping of PB2MP-g-PVDF membranes. With respect to real water analysis, uranyl interference was investigated in presence of Zn(II), Ni(II), Mg(II), Cu(II), and Fe(II) at 10-fold excess concentrations (see SI-1). Apart from Mg(II), U(VI) was found interfering with all metal ions studied. Uranyl is thought to bind to phosphate group of PB2MP. The pKa of phosphate diester is 1.5, thus two types of interaction are expected: ion-exchange with dissociated P–O^−^ and coordination with P=O as shown in Fig. 3.

One of the possible reasons of the uranyl reduction peak decrease at −0.5 V in SW-CSV voltammograms (see SI-1) is a competitive sorption. Such divalent cations may have less sterical constraints to be coordinated by the phosphate group of PB2MP and thus hinder uranyl preconcentration. Change of uranyl speciation cannot also be excluded as it affects uranium in solution before being sorbed and as a result may lead to lower uranyl reduction peak. The cyclic voltammetry study of overall redox reactions of uranyl occurring on a membrane-electrode and SW-CSV parameter optimization were already reported^4^. To better understand which mechanism of uranyl sorption by PB2MP-g-PVDF membranes takes place, one need also to explore other analytical techniques to overcome the SW-CSV sensitivity limit.

### Uranyl sorption

Langmuir, Freundlich and Temkin are the most widely used two-parameter isotherm models for describing metal ion sorption behavior (for model equations and parameters, see SI-3). Among them, Langmuir isotherm model allows to directly get the physical parameters governing the sorption process such as binding affinities of adsorbates *b* and the maximum equilibrium sorption capacity *q*_*max*_. The mathematical representation for a single solute sorption is

5 $${q}_{eq}=\frac{{q}_{max}b{c}_{eq}}{1+b{c}_{eq}}$$

where *q*_*eq*_ is the equilibrium sorption capacity (mg g^−1^ or mmol g^−1^), *i.e*. mass of retained species at equilibrium per mass of membrane, *q*_*max*_ is the saturated monolayer sorption capacity (mg g^−1^ or mmol g^−1^), *b* is the sorption equilibrium constant (L mol^−1^) and *c*_*eq*_ is an equilibrium (final) concentration (mg L^−1^ or mmol L^−1^).

For uranyl sorption, PB2MP-g-PVDF membranes, initially weighted (*m* ≈ 6.40 mg), were placed into aqueous solutions (*V* = 100 mL) of various U(VI) concentrations (*c*_0_ from 0 to 658 ppb), at pH 7. All experiments were performed at room temperature (20 ± 5° C) during 17 hours of sorption in stirred solutions. The concentration before and after sorption were analyzed by ICP-MS previously calibrated. The masses of retained U(VI) at equilibrium per gram of the membrane, *q*_*eq*_, were calculated using equation (6).

6 $${q}_{eq}=\frac{({c}_{0}-{c}_{eq})V}{m}$$

where *c*_0_ and *c*_*eq*_ are initial and equilibrium concentrations of U(VI) in the solution (mg L^−1^), respectively; *V* is a volume of solution (L), *m* is the mass of dry PB2MP-g-PVDF membrane (g).

**Figure 4:**
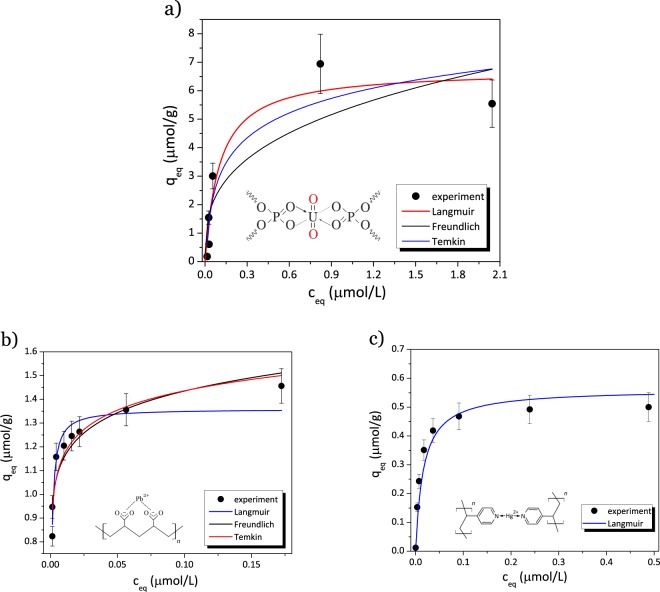
Non-linearised Langmuir, Freundlich and Temkin models applied to experimental metal ion sorption isotherms at liquid-solid interface: (**a**) U(VI) sorption by PB2MP-g-PVDF membranes; (**b**) Pb(II) sorption by PAA-g-PVDF membranes and (**c**) Hg(II) sorption by P4VP-g-PVDF membranes.

Langmuir isotherm model gave the best correlation coefficient (Fig. 4a), the closest *q*_*max*_ and *b* values for non-linearised and linearised plots (Table 1). A monolayer sorption is thus favored.

**Table 1:** Sorption isotherm model parameters of U(VI) by B2MP-g-PVDF membranes.

Model	Langmuir			Freundlich			Temkin		
	*q*_*max*_ (mol g^−1^)	*b* (L mol^−1^)	*R*^2^	*K*_*F*_	*n*	*R*^2^	*K*_*T*_	*b*_*T*_	*R*^2^
No linearisation	6.73 ⋅ 10^−6^	9.85 ⋅ 10^6^	0.89	4.95 ⋅ 10^−4^	3.05	0.71	1.05 ⋅ 10^8^	1.96 ⋅ 10^9^	0.84
Linearisation	6.00 ⋅ 10^−6^	1.15 ⋅ 10^7^	0.97	1.00	1.76	0.60	1.04 ⋅ 10^8^	7.93 ⋅ 10^5^	0.84

In order to better understand which mechanism inside the nano-confined environment was responsible for U(VI)..PB2MP-g-PVDF complexation, we have chosen two other functionalities representing different pre-concentration mechanisms to compare with. Sorption experiments were thus performed on three different sorbing systems representing different metal ion preconcentration effects (Fig. 4). Indeed, the versatility of radiation grafting technique allows any vinyl (or allyl) monomer to be grafted inside the nanopores of track-etched PVDF membranes through remaining radiolytically produced species, *i.e*. radicals according to equations (1)–(4). These systems were: PB2MP-g-PVDF, PAA-g-PVDF, and P4VP-g-PVDF membranes for U(VI), Pb(II), and Hg(II) sorption, respectively. The latter two systems present known metal complexation mechanisms and may serve as sorption models. It is worth mentioning that PB2MP-g-PVDF exhibited a weak affinity for Pb(II) and Hg(II) (see SI-4).

Polyacrylic acid (PAA) and poly(4-vinylpyridine) (P4VP) grafted membranes were fabricated as previously reported^14,15^. The carboxyl group of PAA (-COOH) is a weak acid and partially dissociates in water (pKa is 4.5^18^) possessing pure electrostatic interaction with dissolved metal ions. The metal-carboxylate interaction therefore can be expressed as follows:

7 $${\rm{R}}-{{\rm{C}}{\rm{O}}{\rm{O}}}^{-}+{{\rm{M}}}^{2+}\rightleftharpoons {\rm{R}}-{{\rm{C}}{\rm{O}}{\rm{O}}{\rm{M}}}^{+}+{{\rm{H}}}^{+}$$

8 $${\rm{R}}-{{\rm{C}}{\rm{O}}{\rm{O}}}^{-}+{\rm{R}}-{{\rm{C}}{\rm{O}}{\rm{O}}{\rm{M}}}^{+}\rightleftharpoons {({\rm{R}}-{\rm{C}}{\rm{O}}{\rm{O}})}_{2}\,{\rm{M}}\,$$

In such a system (Fig. 4b), indirect interaction of sorbed species, or sorbed layer re-organization, cannot be excluded. Therefore, Freundlich and Temkin isotherm models appeared to better describe the sorption of lead by PAA-g-PVDF membranes than Langmuir isotherm model at high concentrations (after exceeding 0.05 *μ*mol.L^−1^).

Unlike, when chelatant polymers are expected to act as ligands, namely P4VP and PB2MP, Hg(II) and U(VI) uptakes should be limited to the number of available sorption sites. This hypothesis was confirmed by our experimental results, for which Langmuir isotherm model gave the best fit (Fig. 4a–c).

At the investigated pH range, non-ionized P4VP functional groups can only coordinate with Hg(II) excluding any electrostatic interaction with the metal ions, and once sorbed, no interaction between sorbed species occurred. The Hg(II)^..^P4VP-g-PVDF complexation is known to be a strong coordinate bonding through a lone pair shared with nitrogen atoms or *π*-bonding of pyridine rings.

The U(VI) sorption mechanism onto PB2MP, however, may differ from that of Hg(II) onto P4VP, since phosphate groups of PB2MP possess both ion-exchange and coordination interaction possibilities with ions owing to its chemical structure at studied pH. Although the Langmuir isotherm model proved to be the most appropriate for describing the experimental data among considered two-parameter isotherm models, it nevertheless may be more complex than a simple monolayer sorption. To strengthen the latter discussion, Langmuir sorption equilibrium constant *b* was extracted from the fit in the dilute regime where Langmuir model was found appropriate for the three studied systems. *b* represents metal ion binding affinity to the given sorption sites present in the nanopores and was compared between the different complexation systems as follows: *b*_Pb(II)-PAA_(1.24 ⋅ 10^6^ L mol^−1^) < *b*_U(VI)-PB2MP_(9.850 ⋅ 10^6^ L mol^−1^) < *b*_Hg(II)-P4VP_(7.0 ⋅ 10^7^ L mol^−1^). The equilibrium constant for U(VI)^..^PB2MP-g-PVDF complexes was found to be almost ten times greater than that of Pb(II)^..^ PAA-g-PVDF complexes and seven times lower than that of Hg(II)^..^P4VP-g-PVDF complexes. This ascending order reveals a transition from pure electrostatic interaction to metal-ligand coordination. U(VI)^..^PB2MP complexation character is in between.

### Proposed uranyl sorption mechanism

**Figure 5:**
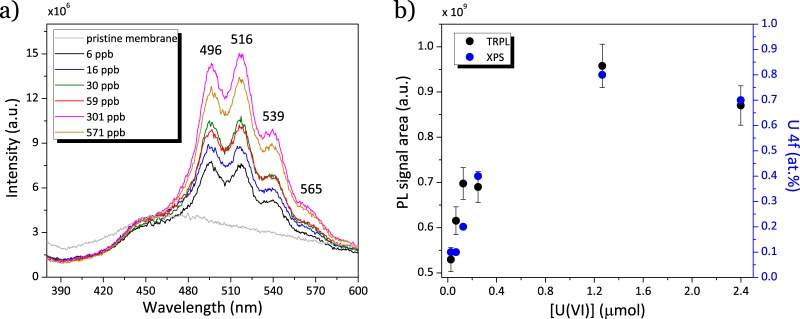
TRPL measurements of uranyl spiked PB2MP-g-PVDF membranes at neutral pH and room temperature: (left) emission spectra; (right) PL signal area correlation with U 4f by XPS.

To better understand the uranyl retention and its sorption mechanism inside the membrane porosity, one need to know if the U(VI) uptake by PB2MP functional groups implies a change in its speciation. The photoluminescence properties of uranyl are known to be strongly dependent on its coordination environment. Time-resolved photoluminescence (TRPL) measurements have been thus performed on uranyl spiked membranes to find out its mechanism of complex formation. Figure 5a depicts emission spectra of uranyl spiked PB2MP-g-PVDF membranes at an excitation wavelength of *λ*_*ex*_ = 355 nm. The broad emission band at 510 nm with well-resolved vibronic peaks with maxima at 496, 516, 539 and 565 nm is a uranyl characteristic emission band^9^ accompanied by a wide emission band (430–550 nm) of non-sorbed PVDF membrane^19,20^. TRPL spectra exhibit a profile which is a clear signature of uranyl in its hexavalent state, *i.e*. U(VI). It demonstrates that the trapping inside the PB2MP-g-PVDF nanoporous membranes did not change the ion speciation. As can be seen from the Fig. 5b, the increase of U(VI) concentration up to approximately 100 ppb (0.42 *μ*mol.L^−1^) results in a nearly linear increase of PL signal area.

However, at higher U(VI) concentrations, the PL signal area levels off confirming the formation a monolayer of adsorbate, where membrane saturation occurs. The amount of retained uranyl correlates well with relative atomic concentration of uranium acquired by XPS (U 4f peak, see SI-2).

**Figure 6:**
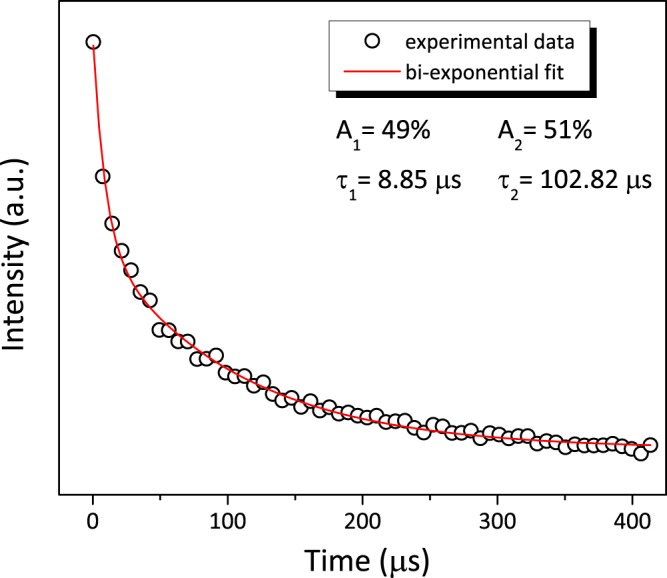
Fluorescence decay of uranyl sorbed PB2MP-g-PVDF membranes.

 Figure 6 displays the fluorescence decay curve of 500 ppb U(VI) sorbed PB2MP-g-PVDF membrane at *λ*_*ex*_ = 355 nm.

The decay was fitted using a bi-exponential function using equation (9).

9 $$I(t)={I}_{0}+{I}_{1}{e}^{-\frac{t}{{\tau }_{1}}}+{I}_{2}{e}^{-\frac{t}{{\tau }_{2}}}$$

where *I*_0_, *I*_1_, and *I*_2_ are scalar values acquired from the data fitting, *t* is time (*μ*s), *τ*_1_ and *τ*_2_ are decay times for exponential components. The resulting parameters are shown in the Fig. 6. The average life-time from the bi-exponential fitting was evaluated using equation (10).

10 $$ < \tau  > =\frac{{I}_{1}{\tau }_{1}+{I}_{2}{\tau }_{2}}{{I}_{1}+{I}_{2}}$$

 < *τ* > was equal to 54 *μ*s. Bi-exponential decay mathematical function was found to better fit experimental data than mono-exponential one. The physical meaning of such a result may be the co-existence of different environments of sorbed uranyl ions. As aforementioned, there are potentially two types of interaction responsible for uranyl retention which may be expected in PB2MP grafted membranes: ion-exchange and coordination. In the literature, for ${UO}_{2}^{2+}$ electrostatic interaction with weak acids at pH > 3.25, Dubolazov *et al*.^21^ reported the suppression of relative luminescence intensity and associated this phenomenon to the appearance of uranyl hydroxide species. In aqueous solutions, hydrolyzed uranyl ions are effectively known to form various complexes, such as [(UO_2_)(OH)]^+^, [(UO_2_)_2_(OH)_2_]^2+^, [(UO_2_)3(OH)_5_]^+^, and (UO_2_)(OH)_2_ (see SI-5). At the light of this observation, the ion-exchange interaction for UO${}_{2}^{2+}$ appears unlikely. Thus, one can consider only coordination with P=O to be responsible for uranyl retention inside the nanopores of PB2MP-g-PVDF membranes, which is in coherence with sorption isotherm results. In our system, the fast life-time (*τ*_1_ = 8.8 *μ*s) can be attributed to uranyl ions binded to only one of the oxygen atoms of PB2MP phosphate group with the rest of uranyl ion coordination possibilities occupied with water molecules. While longer life-time (*τ*_2_ = 102.8 *μ*s) can be assigned to the substitution of water molecules in favor of more effective uranyl coordination with at least two P=O binding contributions, resulting in the U(VI)^..^PB2MP complex stabilization. Almazan-Torres *et al*.^22^ reported two uranyl complexes formed onto zirconium oxophosphate (Zr_2_O(PO_4_)_2_) with Zr-O and P-O surface sites with corresponding life-time values of 20 and 80 *μ*s. Very recently, life-time values of *τ*_1_ = 13.4 *μ*s and *τ*_2_ = 73.2 *μ*s for similar U(VI) complexes on acrylic resin partially composed of PB2MP were also reported^23^. These data are in relative good agreement with our results. The higher < *τ*_2_ > in our work should be thanks to the peculiar geometry of nanoporous sorption system. It suggests that the PB2MP grafted nanopores provide a confinement that facilitates uranyl coordination reactions. Such effect of the confinement is in agreement with uranium sorption reactions already reported^24,25^.

**Figure 7:**
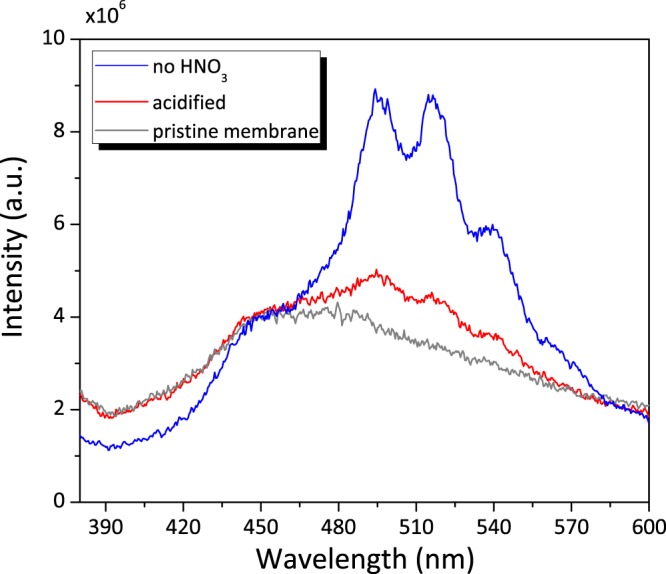
Emission spectra of uranyl spiked PB2MP-g-PVDF with and without 2% HNO_3_, [U(VI)] = 25 ppb.

The efficiency of U(VI) sorption should be pH dependent due to the uranyl speciation (SI-5). Indeed, uranyl solutions for membrane sorption prepared with addition of 2% HNO_3_ caused a significant decrease of characteristic uranyl PL emission band intensity (Fig. 7). The desorption of uranyl ions under acidic pH was previously reported^26^. It allows the release of the uranyl ion and to recycle the membrane for further U(VI) trapping and detection.

## Conclusion

Uranyl sorption behavior of recently developed BP2MP-g-PVDF nanoporous membranes was characterized using Langmuir, Freundlich and Temkin isotherm models. Uranyl sorption obeyed Langmuir isotherm model pointing out that uranyl sorption is limited to a monolayer. Uranyl binding affinity towards PB2MP-g-PVDF was compared with binding affinities of lead and mercury towards respectively PAA and P4VP grafted membranes and was found to have an intermediate characteristics between those of ion-exchange and coordinate functional polymers.

TRPL measurements were performed to better understand the uranyl sorption mechanism inside PB2MP grafted nanopores. The revealed U(VI) characteristic emission band was found increasing almost linearly with increase of U(VI) concentration up to approximately 100 ppb. Whereas at higher U(VI) concentrations, the PL signal area leveled off confirming the sorption isotherm results. Kinetics measurements were fit by bi-exponential decay function exhibiting two decay times, *τ*_1_ and *τ*_2_, which were attributed to the co-existence of U(VI)-phosphate complexes. The fast life-time (*τ*_1_ = 8.8 *μ*s) can be attributed to uranyl ions binded to only one of the oxygen atoms of PB2MP phosphate group, while longer life-time (*τ*_2_ = 102.8 *μ*s) can be assigned to the substitution of water molecules in favor of more effective uranyl coordination with at least two P=O binding contributions. The higher U(VI)^..^PB2MP-g-PVDF complex stabilization should be due to the nanoporous nature of our sorption system. The presented results suggest that the PB2MP grafted nanopores provide a confinement that facilitates uranyl coordination reactions. Effect of pH on uranyl sorption was also pointed out showing a good retention at circumneutral pH and a U(VI) release at strong acidic pH.

It is worth mentioning that the limit of detection for U(VI) adsorbed by PB2MP-g-PVDF membranes using TRPL was as low as 5 ppb (see SI-6). The limit of detection for U(VI) in water by stripping voltammetry was 17 ppb. TRPL technique may consequently be considered as a serious alternative to stripping voltammetry for U(VI) trace detection.

## Supplementary information

Supplementary Information.
