# Supplementary material for: An uranyl sorption study inside functionalised nanopores
Source: Sci Rep. 2020 Apr 1;10:5776. doi: 10.1038/s41598-020-62792-4 (PMC7113287; doi:10.1038/s41598-020-62792-4)
Supplement: Supplementary file 1 — Supplementary Information. [file 41598_2020_62792_MOESM1_ESM.pdf]

Supplementary information

An uranyl sorption study inside functionalised nanopores

U. Pinaeva, N. Ollier, O. Cavani, E. Balanzat, M. Al-Sheikhly, T. L. Wade, M.-C. Clochard

SI-0

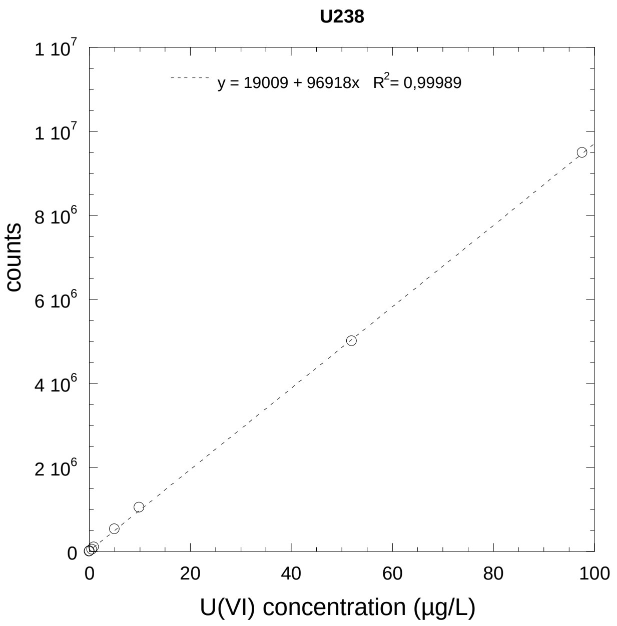

Figure 1: ICP-MS U238 calibration.

SI-1

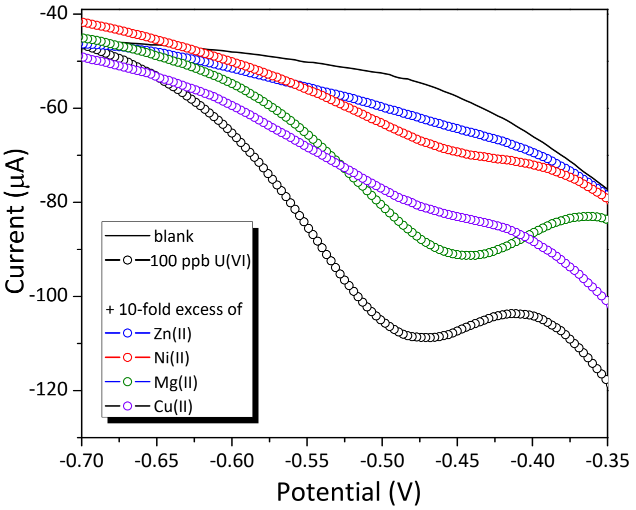

Figure 2: Uranyl interference with Zn(II), Ni(II), Mg(II), and Cu(II) [Pinaeva et al., Reac. and Func. Polym. 142(2019), 77-86].

SI-2

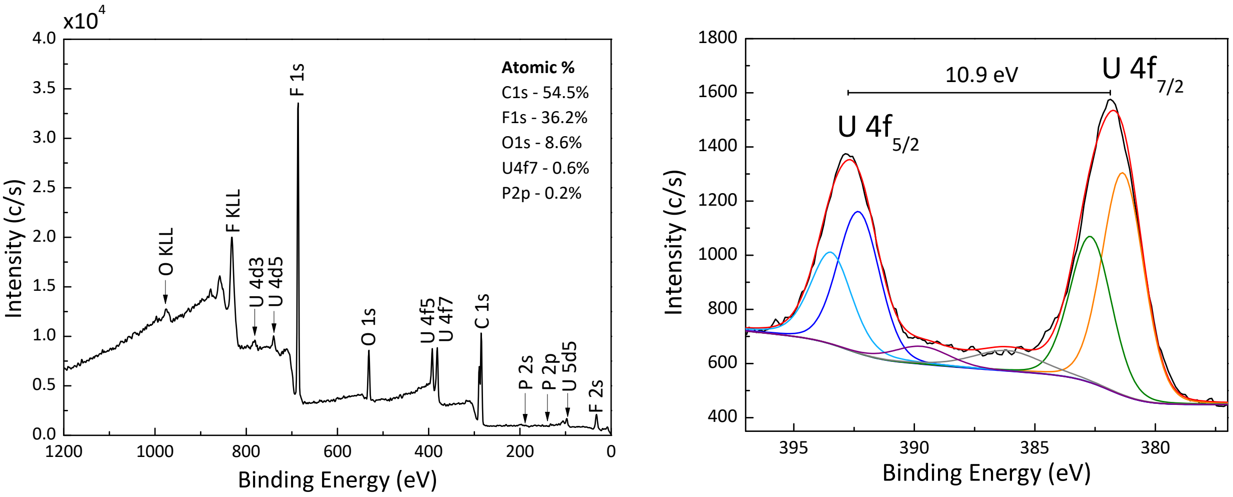

(a) Survey spectrum.

(b) High-resolution spectrum of U 4f peak.

Figure 3: Typical XPS spectra of uranyl loaded PB2MP-g-PVDF membranes at neutral pH and room temperature.

SI-3

Langmuir (1918), Freundlich (1906) and Temkin (1941) are the most widely used two-parameter isotherm models for describing metal ion sorption behavior. Langmuir isotherm model is based on the assumption that binding sites are homogeneously distributed over the adsorbent surface. These binding sites have the same affinity for sorption of a single molecular layer. This model assumes that there is no interaction between adsorbed molecules. The mathematical representation for a single solute sorption is

$$q_{eq} = \frac{q_{max} b c_{eq}}{1 + b c_{eq}} \tag{1}$$

where q_{eq} is the equilibrium sorption capacity (mg g^{-1} or mmol g^{-1}), *i.e.* mass of retained species at equilibrium per mass of membrane, q_{max} is the saturated monolayer sorption capacity (mg g^{-1} or mmol g^{-1}), b is the sorption equilibrium constant (L mol^{-1}) and c_{eq} is an equilibrium (final) concentration (mg L^{-1} or mmol L^{-1}).

Freundlich isotherm model is an empirical equation describing non-ideal, reversible sorption on the basis of an assumption of the surface energetic heterogeneity. It is often applied to multilayer sorption. This model assumes that as the adsorbate concentration increases so does the concentration of adsorbate on the adsorbent surface and can be expressed as

$$q_{eq} = K_F {c_{eq}}^{\frac{1}{n}} \tag{2}$$

where K_F (mol g^{-1})(mol L)$^{-1/n}$ is a constant characterizing the sorption capacity and n (dimensionless) is a constant depicting the sorption intensity.

Temkin isotherm is based on the assumption that heat of sorption would decrease linearly with the increase in coverage of the adsorbent. Similarly to Freundlich model, it is often applied to describe sorption on heterogeneous surface:

$$q_{eq} = \frac{RT}{b_T} ln(K_T \cdot c_{eq}) \tag{3}$$

where K_T is the equilibrium binding constant corresponding to the maximum binding energy, b_T is the Temkin isotherm constant, T is the absolute temperature (K), and R is the ideal gas constant. For all the models presented, the constants can also be determined from the linearised plots.

SI-4

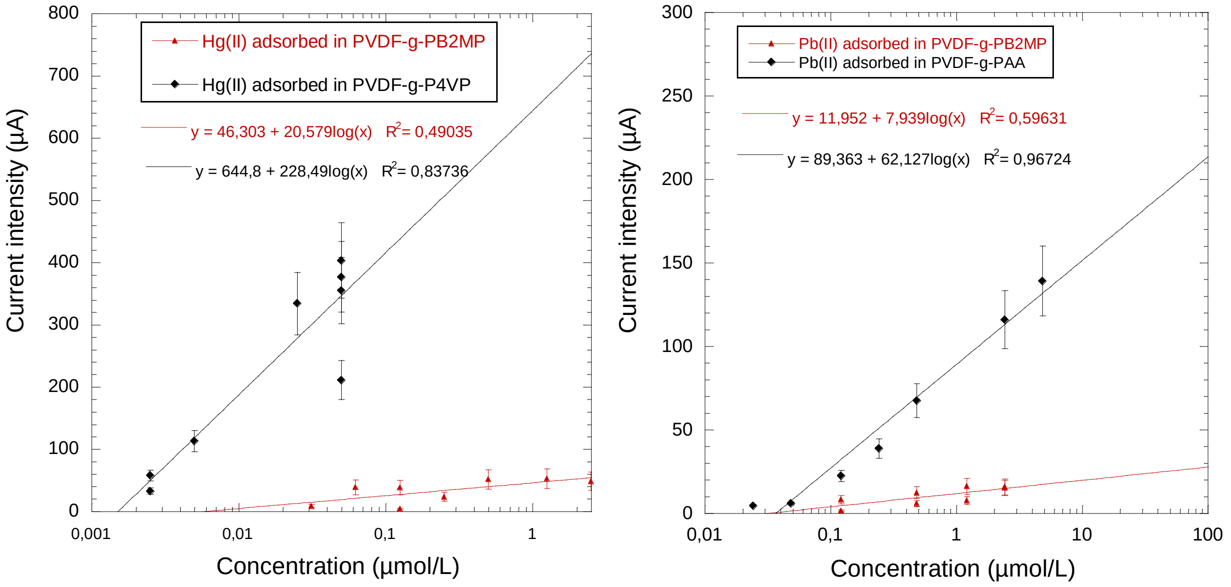

Figure 4: Left: Hg(II) sorption calibration with PB2MP-g-PVDF and P4VP-g-PVDF nanoporous membranes; Right: Pb(II) sorption calibration with PB2MP-g-PVDF and PAA-g-PVDF nanoporous membranes.

SI-5

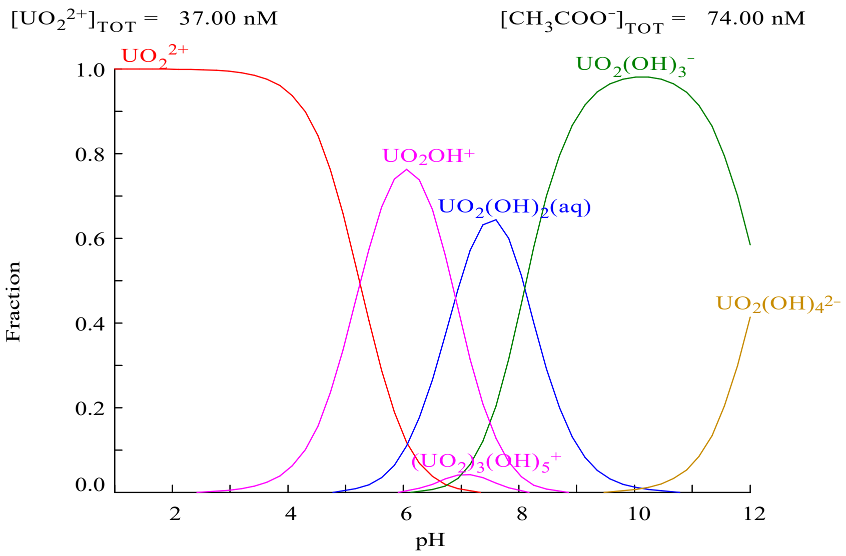

Figure 5: Uranyl speciation as a function of pH in deionized water, $[U(VI)]$=10 ppb (simulated by Hydra-Medusa Equilibrium Diagrams).

SI-6

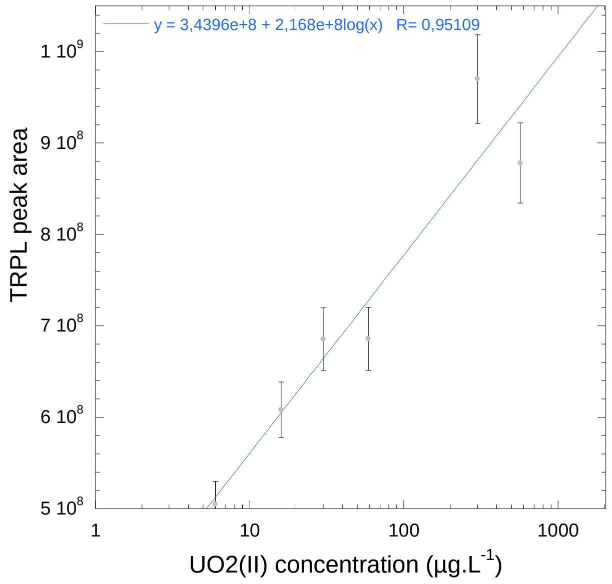

Figure 6: U(VI) LOD determination from TRPL measurements against U(VI) concentration.
